# Supplementary material for: Optimal design and operation of Photovoltage distributed generators and shunt compensators for the Vietnam alternative current distribution network to reduce annual energy loss
Source: PLoS One. 2026 Mar 2;21(3):e0342402. doi: 10.1371/journal.pone.0342402 (PMC12952659; doi:10.1371/journal.pone.0342402)
Supplement: Attachment — Submitted filename: PLO-Responses to Reviewers-R1.docx [file pone.0342402.s004.docx]

**RESPONSES TO REVIEWERS**

**The authors really appreciate the editor and reviewers to give us chances to revise the manuscript for publication. The authors are grateful to the reviewers for giving us useful comments and suggestions. The paper is long, and the understanding of the paper might take too much time for the reviewers. So, the authors have considered all comments carefully and tried to improve the existing shortcomings. In the current form of the manuscript, changes have been marked in blue, and responses to comments are listed below. Please take the time to see the revised manuscript. Thank you very much.**

**RESPONSES TO COMMENTS OF REVIEWER #1**

***Reviewer #1****: Overall, the paper addresses a relevant problem and provides a comprehensive study of optimal placement and operation of PVDGs and SCBs using FGO and AOO on three distribution networks, including a 55-node feeder in Vietnam.*

The two-phase structure (design versus operation) and the comparison of AOO against both FGO and a large set of existing algorithms give the work some depth, and the numerical results are clearly in favor of AOO.

To me, the narrative sometimes feels dense and a bit repetitive, and metaheuristics are not convincing enough. Please see my comments below:

**Comment 1**. In terms of logical flow, the Introduction is quite long and the transition from the broad literature review to the specific scope of “two-phase design and operation” is not always smooth. I have two suggestions here:

- For example, Section 1.3 lists “novelties” and then “main contributions” that overlap and could be merged into a more focused, shorter list, directly tied to what is actually done in Sections 2–4.
- You might also help readers by explicitly reminding them at the start of Section 4 how the design phase (peak-hour TAPL, equations (1)–(2)) and the operational phase (24-hour and quarterly energy-loss evaluation) fit together in the overall story.

Response to Comment 1:

Thank you very much for your comment!

Based on the reviewer’s first suggestion, the novelites and the main contribution have been merged using a short list as follows:

“ *The novelties and the main contributions of the whole study can be emphasized by the following terms:*

- *Successfully apply two novel meta-heuristic algorithms to optimize the placement of PVDGs and SCBs to different ACDNs configurations, including the IEEE 69, 85-node, and a practical one in Vietnam with 55 nodes, for power loss reductions with constant load demand, considering different configurations of DGs*
- *Presents a detailed evaluation and comparison of the effectiveness of the applied algorithms in addressing the considered problem across various criteria. Based on the results and achievements, AOO demonstrates itself to be a powerful and robust search algorithm compared to FGO and the previous ones.*
- *Presents various strategies for placing PVDGs and SCBs to reduce TAPL, with a detailed evaluation of their practical effectiveness in supporting the design process of the Vietnam ACDN, which has 55 nodes.*
- *For each of the placement strategies of PVDGs and SCBs on the Vietnam practical ACDN as mentioned above, a 24-hour operational simulation is conducted with load demand variation and dynamic supplied power from PVDGs to provide a detailed view of the actual amount of active power loss reduction at e ach hour.*
- *The study also presents a seasonal evaluation of energy loss reduction, based on average daily load profiles and PVDG output on an average day of the twelve months. This analysis highlights the influence of seasonal variations on the effectiveness of the proposed solutions* ”

As per the second suggestion from the reviewer regarding the explicitness of the entire work conducted in the research, the additional explanation regarding the use of Equations (1) and (2) in Subsection 2.1 is given, explaining which one is utilized to serve the planning phases and which one is applied to support the operational phase as follows:

*“Note that Equation (1) is mainly use to serve the designing phase finding the highest power loss value with peak demand power and the optimal placement of DGs, while Equation  (2) is applied to determined the energy loss value in operational phase at each hour of a day, then the energy loss of a month, a year will be subsequently determined”* .

Furthermore, an additional reminder regarding the execution of the design phase and operational phase is also added below the case description in Section 4 for better understanding and to be followed by the reader as follows:
“** Note that the results of all the cases mentioned in i) and ii) will be delivered subsections 4.1 to 4.3 to serve the designing phase with the main objective function shown in Equaiton (1) of subsection 2.1 and to demonstrate the capability of the applied algorithms, and then only the best applied algortihm will be reapplied in the operational phase in which only the optimal designs achieved in all the cases in ii).*”

**Comment 2**. Regarding the model design, why do you use a current-based formulation instead of power-flow-based expressions?

Response to Comment 2:

Thank you very much for your comment!

1. The Backward and forward sweep method.

Backward and Forward Sweep (BFS) is a tool for solving the power flow problem in a distribution power system [A1].  ***This is a current-based, iterative method*** executed in two stages, including the backward and forward stages. The description of these two stages is given as follows:

- The backward sweep: this stage aims to determine the branch current based on the sum of load current at each node plus other downstream using the Kirchhoff’s law as presented in Equations (1) - (2) belows [A1]:

$$I_{n}=\frac{S_{n}}{V_{n}}$$

(1)

$$I_{nm}=I_{m}+\sum I_{m,linked}$$

(2)

Where, $I_{n}$ is the current amplitude required by load connected to node *n* with *n* = 2, 3, …, $N_{No}$ and $N_{No}$ is the number of node in the given DPN; $S_{n}$ is the apparent power required by load n,; $V_{n}$ is the voltage magnitue at node *n*; $I_{nm}$ is the branch current between node *m* and *n*; $\sum I_{m,linked}$ is The sum of all branch currents flowing to nodes downstream of node *m*.

- Forward sweep: this stage is executed to compute the voltage node from the source (node 1) to the furthest node of the given grid by applying the Kirchhoff’s law [A1]:

$$V_{m}=V_{n}-Z_{mn}\times I_{mn}$$

(3)

Where, $V_{m}$ is the computed voltage at node m; $Z_{mn}$ is the impedance of the distribution line connecting node m and *n* which are represented by resistance (R) and reactance (X).

As mentioned earlier, BFS is the iterative method; hence, there should be a stopping condition to terminate the computing process while the output reaches the intended measurement, normally set by the total deviation of the voltage node denoted by $\Delta V$. According to [A1], when the error reaches a certain value as expected initially, the entire computing process will be terminated, and all branch currents and voltage nodes will be reported. Based on the branch currents, the power loss for each branch and the whole grid will be determined as follows:

$$P_{loss,br}=R_{br}\times I_{Br}^{2}$$

(4)

$$TAPL=\sum_{br=1}^{N_{br}} P_{loss,br}$$

(5)

Where, $P_{loss,br}$ is the power loss on the branch *br* with *br* = 1, 2, …, $N_{br}$ and $N_{br}$ is the number of branches in the given ACDN; $R_{br}$ is the resistance value of the branch br; $I_{br}$ is the branch current determined in the backward stage as described above; $TAPL$ is the total active power loss determined the sum of power loss of all branhes.

2. The Newton-Raphson method

Newton-Raphson (NR) power flow method is also another tool for power flow calculation that mostly applied to solve the power flow problem in transmission power network (TPN) [A2]. Basically, NR is also an iterative method that mostly applies voltage deviation error as the stopping conditions to terminated the computing process. Furthermore, *NR is a power flow based method* which is different from BFS as anslyzed in 1. NR is widely applied to solve the power flow problem in TPN configurations due to its best leverage to the the physical structure of most TPN which highly constructed as loop to improve the reliability and contiounity of supplied power. The determination of power loss while using the NR method is different from BFS method as mentioned earlier. Particularly, the power loss of the transmission line is achieved using the following model [A3]:

$$\sum P_{loss}=V_{n}\sum_{n=1}^{N_{no}} V_{m}\left[ G_{nm}\times\cos\left( \theta_{n}-\theta_{m} \right)+B_{nm}\times\sin\left( \theta_{n}-\theta_{m} \right) \right]$$

(7)

Where, $\theta_{n}$ and $\theta_{m}$ are the voltage phase angle at node *n* and *m*; $G_{nm}$ and $B_{nm}$ is the conductance and susceptanace value taken from the atmittance matrix ($Y_{bus}$) which represented the line parameters. Particularly, the $Y_{bus}$ is determined by:

$$Y_{nm}=G_{nm}+jB_{nm}$$

(8)

Usually, $Y_{bus}$ is a matrix with a size based on the number of nodes in the given transmission power system and the formation of $Y_{bus}$ is also dependent on the line model applied which are T-shape or $\pi$-shape, initially. Particularly, in the considered transmission power system with *n* nodes, the size of the $Y_{bus}$ will be *n* x  *n.* In fact, the power loss in Equation (1) is specified initially as the mismatched power between the two attribute nodes in the given power loss as described in the Jacobian Matrix, and the establishment of this Jacobian matrix is a must in executing the NR method. Normally, the Jacobian matrix is formed by the following terms:

$$\left[ \begin{aligned} \Delta P \\ \Delta Q \end{aligned} \right]=\left[ \begin{aligned} J_{1} J_{2} \\ J_{3} J_{4} \end{aligned} \right]\left[ \begin{aligned} \Delta\theta\\ \Delta\left| V \right| \end{aligned} \right],$$

(9)

Where $\Delta P$ and $\Delta Q$ is the mismatched in active and reactive power; $\Delta\theta$ and $\Delta\left| V \right|$ is the deviation of voltage in phase angle snd magnitude; $J_{1}$ is the sensitive indicator of active power to phase angel; $J_{2}$ is the sensitive indicator of active power on voltage magnitude; $J_{3}$ is the sensitive indicator of reactive power to phase angel; $J_{4}$ is the sensitive indicator of reactive power on voltage magnitude.

3. The selection of BFS over NR and other power-flow-based method to the give problem.

Based on the brief introduction of the BFS and NR for power flow calculation as presented in the first two subsections above, there were clear reasons to lean forward to the BFS to solve the given optimization problems as follows:

- BFS is only based on the Kirchhoff law to achieve the branch current and voltage nodes, significantly less complicated than NR, which requires the selection of a line model and the formation of a Jacobian matrix.
- NR possibly faces the ill-condition caused by the high ratio of R/X, as mentioned in [A4-A5], which makes the method fail to converge. BFS, on the other hand, provides a better capability to deal with the high ratio of R/X, which is mostly the nature of the distribution power grid.
- As presented above in the previous section, the execution of backward and forward stages provides a superiority in tree-shaped or star-shaped configurations of most distribution power systems, unlike the transmission power systems, which are highly reliant on the loop structure.
- The power loss, which is the main objective function of the manuscript, is achieved by the multiplication of current branch and conductor’s resistance, as already presented in Equation (4), is not corrected when applied in the transmission power networks, in which the power loss is determined by Equation (7).

With all the analyses and evaluations mentioned in the three sections above, the authors hope that these proofs have successfully addressed the concerns raised by the reviewer. Additionally, the authors would like to thank the reviewer for providing us with the opportunity to provide an extensive explanation of our work.

**Comment 3**. On the methodology side, the descriptions of FGO and AOO in Section 3 are mathematically detailed but a bit overwhelming. It might be very, very useful to give a short “high-level” explanation after each update rule, e.g., which parts are mainly responsible for global exploration versus local refinement.

Response to comment 3: Thank you very much for your comment!

After carefully considering the reviewer’s comments, the authors agree that the description of the two applied algorithms, including FGO and AOO, regarding their update process may be unclear and not user-friendly. Hence, the descriptions of the update procedures for both FGO and AOO have been carefully revised, along with the additional pseudo-code for each, aiming to provide better clarity and readability to the reader. The authors highly appreciate the reviewer’s comment. Please take a look at the revised manuscript for full details.

**Comment 4.** Why do you use heuristic algorithms? In power system research, they always use MILP/LP to ensure the optimum of the results. You say your design is "optimal," but you are using a heuristic method, which is somewhat contradictory.

Response to comment 4:

Thank you very much for your comment!

First of all, the author would like to provide a brief evaluation of the Mixed-Integer Linear Programming/Linear Programming (MILP/LP) as mentioned by the reviewer and its performance in dealing with various optimization problems, particularly large-scale ones. According to the authors in [A6], MILP/LP is designed to deal with optimization problems with linear characteristics and highly prioritizes the precision of the optimal results over other aspects, such as convergence speed and response times. Additionally, MILP/LP utilizes specific mathematical models derived from a certain process; hence, the application of this method ensures the finding of the optimal solution to the given problem. Furthermore, the authors in [A7-A8] have proven the capability and effectiveness of MILP/LP in solving different optimization problems in practice.

However, the development in engineering has led to the need to solve large-scale, highly complex optimization problems, in which the application of MILP/LP reveals particular drawbacks compared to modern meta-heuristic algorithms. Particularly, the authors in [A9] have indicated the clear superiority of meta-heuristic algorithms in dealing with multi-objective functions in large-scale power systems in terms of solution quality and resilience computing compared to MILP/LP, which struggle to reach feasible solutions. Furthermore, the statistical results provided by [A10] throughout over 100 other studies also indicated that meta-heuristic algorithms are a better-suited method than MILP/LP  due to their capability to deal with the non-convex, large space solution and high complexity optimization problem without requiring a series of mathematical complications.

The supriroity of mordern meta-heuristic compared to MILP/LP comes from the following key factors:

- Meta-heuristic algorithms do not require an exact or complex mathematical model to solve a given optimization problem, which significantly increases their flexibility and adaptability across different domains.
- Meta-heuristic algorithms are mostly the population based-method therefore they provide the capability to assess a large set of solutions simultaneously. This population-based approach makes the search process more efficient than MILP/LP, which typically focuses on determining a single solution at a time.
- Meta-heuristics are designed to highly utilize the processing performance of modern CPUs. This results in a fast response capability when solving large-scale optimization problems that might otherwise take MILP/LP solvers hours of computation with poor efficiency.
- Most meta-heuristic algorithms are designed by mimicking the behavior of animals in nature or simulating physical laws. This makes them simpler to understand and easier to apply to various optimization challenges without requiring deep expertise in linear programming.

Furthermore, Table A1 below will provide generall look regarding the advantage and disadvantage between MILP/LP and meta-heuristic in dealing with optimization problems

Table A1: The comparision on advantage and disadvantage of MILP/LP and meta-heuristic algorithms

on different aspects.

| Aspect | Algorithm | Advantage | Disadvantage |
| --- | --- | --- | --- |
| Optimal degree | MILP/LP | Provides a clear logical mathematical foundation that is surely reaching global optimum to the given optimizaiton problem | Low efficiency in dealing with non-linear complexities, due to the requirement of exact mathematical model. |
|  | Meta-heuristic | Capable of finding high-quality "near-optimal" solutions for complex, non-linear problems. | No mathematical guarantee that the final result is the absolute global optimum. |
| Problem scale | MILP/LP | Highly efficient for small to medium-scale linear problems. | Computation time, and computing complexity might grow exponentially as problem scale increases. |
|  | Meta-heuristic | Offers fast response times for large-scale problems by making using of the paralel processing of the mordern CPUs | Often require multiple runs or parameter tuning to ensure consistency in results. |
| Mathematical modelling | MILP/LP | Standardized framework ensures consistency and rigorous theoretical backing. | Requires deep expertise to formulate strictly linear or integer constraints. |
|  | Meta-heuristic | Flexible and no need for an exact mathematical model.  Can be used as an independent tool that only requires specific outputs from the user. | Algorithm selection and parameter setting are typically a process of trial and error to define the optimal ones. |

 Based on the detailed analyses above, the author hopes that the reviewer will acknowledge the logical reasons for selecting meta-heuristic algorithms to solve the problem presented in the manuscript. Furthermore, the term "optimal" used to evaluate the results of the applied methods in comparison to others is supported by the statistical results of 50 test runs. For the considered optimization problem, these statistical results provide a clear proof of the stability and consistency of the results, and also address the potential concern regarding the contradiction in our conclusion about the optimal degree obtained by the applied algorithms.

**Comment 5**. The choice of algorithmic parameters in Table 1 (e.g., N_pop = 20–40 and I_max = 200–300, with 50 test runs per case) is reasonable, but currently looks ad hoc. Can you justify? One suggestion (optional but nice to have): show convergence curves (best TAPL versus iteration) for at least one case, as well as average CPU times.

Response to comment 5:

Thank you very much for your comment!

The concern of the reviewer regarding the control parameters of the two applied algorithms in Table 1 of the manuscript is entirely reasonable. In fact, population size (*N_pop_*) and Maximum iteration (*I_max_*) are the two crucial settings that directly affect the output of the algorithm. Particularly, the selection of *N_pop_* significantly affects the solution quality and response time for each iteration, while *I_max_* directly affects the solution quality and response time of each independent run. In cases where both *N_pop_* and *I_max_* are set to very high values, the probability of achieving optimal solutions will be higher; however, the entire search process will be significantly more time-consuming. Moreover, the performance difference among the applied algorithms will be largely blurred in comparison. A meta-heuristic algorithm that is considered to be effective and powerful if it can determine optimal results with low settings of the initial control parameters and is less time-consuming. In fact, there were no standard methods for selecting the optimal control parameters of a certain algorithm for any optimization problem. For most cases, the settings of *N_pop_* and *I_max_* are highly dependent on the developer's experience and the complexity of the given optimization problem.

Nevertheless, the selection based on the developer's experience does not mean taking random values or settings to very high values for the high-complexity optimization problem. In fact, the selection of *N_pop_* and *I_max_* presented in Table 1 of the manuscript is the result of a series of experiments. Taking Case 1 implemented on the IEEE 69-node DPN as an example, the determination of *N_pop_* = 20 and *I_max_* = 200 is the justification for the following tests:

Table A2: The testing of *N_pop_* with fixed *I_max_* for AOO

| *N_pop_* | 5 | 10 | 15 | 20 | 25 |
| --- | --- | --- | --- | --- | --- |
| *I_max_* | 200 | 200 | 200 | 200 | 200 |
| TAPL | 71.665 | 69.693 | 69.461 | 69.426 | 69.426 |

Table A3: The testing of *I_max_* with fixed *N_pop_* for AOO

| *N_pop_* | 20 | 20 | 20 | 20 | 20 |
| --- | --- | --- | --- | --- | --- |
| *I_max_* | 50 | 100 | 150 | 200 | 250 |
| TAPL | 71.272 | 69.787 | 69.693 | 69.426 | 69.426 |

Based on the above results in Tables A1 and A2, the settings of *N_pop_* = 20 and *I_max_* = 200 are the affordable values for this case. Besides, the execution of 50 test runs for each case is aimed at ensuring the optimal results found by the algorithms for each case. It is also one of the important factors for evaluating the stability of the applied algorithm. The selection of 50 test runs per case provides a balance in computing time, and also offers an overall look at the performance of a given meta-heuristic algorithm. For each case study presented in the manuscript, these experiments are repeated until affordable settings are found. However, the addition of these experiments to the manuscript will unintentionally increase its overall length and may lead readers to move away from the primary focus; hence, these contents are not presented in the main text.

Additionally, the convergence curves of the two applied algorithms in Cases 1 and 2, executed in the first two DPN configurations (including the IEEE 69- and 85-node), are added in the revised manuscript along with the computing times. Please review our revised manuscript for further details.

REFERENCES

[A1] Díaz, G., Gómez-Aleixandre, J., & Coto, J. (2015). Direct backward/forward sweep algorithm for solving load power flows in AC droop-regulated microgrids. *IEEE Transactions on Smart Grid*, *7*(5), 2208-2217.

[A2] Sun, D. I., Ashley, B., Brewer, B., Hughes, A., & Tinney, W. F. (2007). Optimal power flow by Newton approach. *IEEE Transactions on Power Apparatus and systems*, (10), 2864-2880.

[A3] Duong, T. L., Duong, M. Q., Phan, V. D., & Nguyen, T. T. (2020). Optimal Reactive Power Flow for Large‐Scale Power Systems Using an Effective Metaheuristic Algorithm. *Journal of electrical and computer engineering*, *2020*(1), 6382507.

[A4] Tostado-Véliz, M., Kamel, S., & Jurado, F. (2021). Power flow solution of Ill-conditioned systems using current injection formulation: Analysis and a novel method. *International Journal of Electrical Power & Energy Systems*, *127*, 106669.

[A5] Muruganantham, B., Gnanadass, R., & Padhy, N. P. (2016, December). Performance analysis and comparison of load flow methods in a practical distribution system. In *2016 National Power Systems Conference (NPSC)* (pp. 1-6). IEEE.

[A6] González, M., López-Espín, J. J., & Aparicio, J. (2020). A parallel algorithm for matheuristics: A comparison of optimization solvers. *Electronics*, *9*(9), 1541.

[A7] Sattar, D., & Matrawy, A. (2019). Optimal slice allocation in 5G core networks. *IEEE Networking Letters*, *1*(2), 48-51.

[A8] Guo, Y., Yao, J., Su, R., Ling, K., Han, B. S., & Wong, H. Y. A. (2024, June). A milp model for conflict-free routing problem with partial charging in multiple-agv system. In *2024 IEEE 18th International Conference on Control & Automation (ICCA)* (pp. 749-754). IEEE.

[A9] Morquecho, E. G., Torres, S. P., Astudillo-Salinas, F., Ergun, H., Van Hertem, D., Castro, C. A., & Blum, C. (2023). Comparison of an improved metaheuristic and mathematical optimization based methods to solve the static ac tnep problem. *IEEE Transactions on Power Systems*, *39*(2), 3240-3256.

[A10] Sedghi, M., Ahmadian, A., & Aliakbar-Golkar, M. (2016). Assessment of optimization algorithms capability in distribution network planning: Review, comparison and modification techniques. *Renewable and Sustainable Energy Reviews*, *66*, 415-434.

**RESPONSE TO COMMENTS OF REVIEWER #2**

***Reviewer #2****: This study focuses on Vietnam's AC distribution network and uses two metaheuristic algorithms, FGO and AOO, to optimize the configuration of Photovoltaic Distributed Generators (PVDGs) and Shunt Capacitor Banks (SCBs) for reducing annual energy loss. Although it has a clear application scenario, there are multiple key flaws in research design, case logic, and data presentation, resulting in insufficient scientificity and rigor.*

**Comment 1**. In the case of the 55-node power grid in Vietnam, the reactive power injection of SCBs in Case 4 (2070, 2250, 1230 kVar) is much higher than that in other cases, and the engineering feasibility of this configuration and its impact on power grid harmonics are not explained.

Response to coment 1:

Thank you very much for your comment!

As addressed by the reviewer, an additional explanation has been added to provide more detailed information about the results regarding the higher amount of reactive power supplied to the grid. Particularly, the additional content is focused on three aspects, including the explanation of why Scenario 4 requires more reactive power injected to the grid than other cases, the feasibility of the combination of PVDGs and SCBs in Scenario 4, and the additional evaluation regarding harmonic management on the grid with high penetration of PVDGs is also added to the revised manuscript. The author hopes that the reviewer will find that the additional content satisfies the concerns initially raised. Additionally, the additional content is blue in the revised manuscript and can be found at *“The observation in Table 6 … and the capital investment.”* Please take a look!

**Comment 2**. In the operational phase, only the average daily data of the first month of each quarter is selected, without considering the load fluctuation within the season and the photovoltaic output change under extreme weather, resulting in insufficient data representativeness.

Response to comment 2:

Thank you very much for your comment!

After reconsidering the reviewer’s concern, the author totally agrees that the selection to simulate energy loss for only the first month of each quarter in the year does not fully leverage the overall look of the results due to the lack of power demand variation and radiation changes throughout the year, reducing the justification and reliability of the final conclusions. Hence, to address the reviewer’s concern, the simulations of energy loss for the twelve months of a year in all four cases have been executed, and the additional results have been added to the revised manuscript. Particularly, Figures 13, 14, and 15 have been modified and renamed as Figures 16, 17, and 18, respectively, to extensively complement the display of energy loss for 12 months, the reduced energy loss for 12 months, and the voltage nodes for 12 months in Scenario 1. Similarly, Figures 17, 18, and 19 are now Figures 20, 21, and 22, respectively, showing the changes in Scenario 2. Next, Figures 21, 22, and 23, which present the changes in Scenario 3, are now Figures 24, 25, and 26. Moreover, Figures 25, 26, and 27, which provide an overview of the changes in Case 4, are now Figures 28, 29, and 30. Please review our revised manuscript for further details.

The authors hope that the reviewer will find our additional simulation and results helpful in improving the sufficiency and the overall justification of our study.

**Comment 3**. When comparing AOO with other algorithms, only the numerical difference of TAPL is listed, and the comparative analysis of core performance indicators such as algorithm convergence speed and computational complexity is not provided.

Response to comment 3:

Thank you very much for your comment!

By considering the reviewer's concern, the author totally agrees that the current manuscript lacks a performance evaluation of the two applied algorithms, and the computational complexity is not clearly investigated. To address the reviewer's concern, the additional proofs regarding the convergence curves and the computing times of FGO and AOO while solving different cases in the two ACDN configurations, including the IEEE 69 and 85 nodes, have been provided along with detailed analyses. Moreover, the computational complexity is also analyzed by emphasizing the difference of desired variables that need to be optimized in all cases implemented in the manuscript. Particularly, Table 4 is provided to offer a detailed look at the quantity of variables for each case on each ACDN configuration, along with extensive analyses which can be found at *“The outperformance of AOO compared to other previous methods … the overall complexity of Scenario2”*.

The author hopes that the reviewer will find the additional contents useful to improve the overall quality of the whole manuscript. Please take a look at the revised manuscript for more details.

**Comment 4**. In the case, the PVDG penetration rate is set to 25%, 50%, 75%, and 100%, but the basis for selecting this penetration rate range is not explained, and whether it is consistent with the local power grid access standards in Vietnam is not mentioned.

Response to comment 4:

Thank you very much for your thoughfull comment!

As addressed by the reviewer, the extensive evaluation and detailed analyses have been added to the revised manuscript and marker blue. The additional content is highly focused on explaining the logical reasons behind the selection of the four penetration levels of PVDGs for the Vietnam ACDN with 55 nodes, referencing the implementation of previous studies and Vietnamese policies. Furthermore, Figure 14, which shows the voltage values for the four penetration levels, has been added to provide clear evidence of adherence to Vietnamese regulatory requirements. Those additional contents are marked blue in the revised manuscript, and they can be found at *“The execution of the four different penetration levels of PVDGs … and the improvement of technical and control factors.”* Please review our revised manuscript.

**Comment 5**. The constraint conditions do not consider the installation cost and maintenance cost of PVDGs and SCBs, and only take loss reduction as the goal, lacking economic feasibility analysis, which does not meet the actual engineering needs.

Thank you very much for your thoughfull comment!

The authors fully agree with the reviewer that the current manuscript primarily focuses on achieving the optimal energy loss in design and operation. The study lacks detailed evaluations and calculations regarding the installation and maintenance costs of PVDGs and SCB, as well as the economic feasibility of the entire project. In fact, the installation, maintenance, and economic feasibility are the key factors that directly determine the actual implementation in practice. Particularly, if an engineering project cannot demonstrate clear economic potential, the likelihood that the project will remain in the draft stage is high. However, considering these factors in the study required complete changes in problem modelling and simulation results; hence, to partially address the reviewer’s concern, the authors have open a new subsection called *“4.6. The discussion on the economic feasibility of the PVDGs project in Scenario 4”* aimed to provide the detailed calculation for the total of implementing the PVDGs, as shown in Scenario 4 compared to the original configuration where PVDGs is not implemented and then clearly prove the economic feasibility of the entire project by achieving the total accummuated profit after the considered project’s lifetime of $8480854.37.

Please take a look at the new Subsection 4.6 of our revised manuscript.

**Comment 6**. The original topological parameters of the 55-node power grid in Vietnam (such as line impedance and node load characteristics) are not fully presented, making it impossible to verify the authenticity and repeatability of the simulation results.

Response to comment 6:

Thank you very much for your comment!

The authors highly appreciate the reviewer’s concern regarding the lack of data availability for the 55-node power grid in Vietnam, which is an important foundation for executing all the involved simulations and increasing the practicality of the entire research. As commented, the specifications of the power grid regarding line impedance and loads have been added to Table A1 in the Appendix of the revised manuscript. Please take a look!

**Comment 7**. The discussion section does not explain why Case 4 performs worse than Case 2 in the third quarter (July), and fails to conduct in-depth analysis of the reasons combined with the seasonal changes of photovoltaic output and load characteristics.

Response to comment 7:

Thank you very much for your comment!

Firstly, the authors totally agree that the presentation of Figure 32 lacks a detailed evaluation regarding the efficiency of Scenario 4 throughout the four selected months, which are the first month of the four quarters in a year. The absence of a detailed evaluation might cause the misunderstanding or improper perception of the initial message that the author would like to convey to the reader. Secondly, the authors would like to note that in the original version of Figure 32, Scenario 4 performs worse than Scenario 1, not Scenario 2, as shown in Subfigure 32c, as mentioned by the reviewer. After seriously considering the reviewer’s comment, the authors realized that Figure 32 needs to be revised for improved aunderstanding and clarity. Particularly, the energy loss of the four selected months is fully displayed within 24 hours instead of only from 6 to 18 hours in the old version. Moreover, additional analyses have been included in the revised manuscript to provide more detailed explanations regarding the performance of Scenario 4, which is not superior to Scenario 1 in June. Particularly, the analysis focuses on the relationship between the three terms: the load factors, the monthly power output from PVDGs, and the injected reactive power to the grid.

The new Figure 32 and the additional content required have been added to the revised manuscript and are marked in blue. Please take a look!
